# Trends of hospital-based reporting of intracranial neoplasms in Nigeria from 1960 to 2024: A systematic review and pooled analysis of literatures

**DOI:** 10.1093/noajnl/vdaf195

**Published:** 2025-09-02

**Authors:** Kehinde Alare, Samson Adedeji Afolabi, Busayo Adetunji, Chukwunonso Emmanuel Chukwumaeze, Emmanuel Kolawole Oluwumi, Kehinde Abdulazeez Periola, Taiwo Omoniyo, Okikioluwa Odesanya, Precious Adedokun, Stephen Ishola Adedokun, Nenkimun Dirting Bakwa, Joshua Opanike, Eromo Kokogho, Oladunjoye David Olaniyan, Paul Ibukunoluwa Oyediran, James Balogun

**Affiliations:** Bowers Neurosurgical Frailty and Outcomes Data Science Lab, Sandy, Texas; Department of Medicine, Ladoke Akintola University of Technology, Ogbomoso, Nigeria; Department of Medicine, Ladoke Akintola University of Technology, Ogbomoso, Nigeria; Department of Medicine, Ladoke Akintola University of Technology, Ogbomoso, Nigeria; Department of Medicine, University of Nigeria Teaching Hospital, Ituku Ozalla, Nigeria; Department of Medicine, Ladoke Akintola University of Technology, Ogbomoso, Nigeria; Department of Medicine, Federal Teaching Hospital, Ido-Ekiti, Nigeria; Department of Medicine, Ladoke Akintola University of Technology, Ogbomoso, Nigeria; All Saints University School of Medicine, Dominica; Department of Surgery, LAUTECH Teaching Hospital, Ogbomoso, Nigeria; Department of Surgery, LAUTECH Teaching Hospital, Ogbomoso, Nigeria; Division of Neurosurgery, Department of Surgery, Jos University Teaching Hospital, Jos, Plateau, Nigeria; Department of Neurosurgery, National Hospital Abuja, Abuja, Nigéria; Department of Medicine, Ladoke Akintola University of Technology, Ogbomoso, Nigeria; Department of Medicine, Federal Teaching Hospital, Ido-Ekiti, Nigeria; Department of Surgery, LAUTECH Teaching Hospital, Ogbomoso, Nigeria; Department of Medicine, Ladoke Akintola University of Technology, Ogbomoso, Nigeria; Department of Neurosurgery, University College Hospital, Ibadan, Nigeria; Division of Neurosurgery, Department of Surgery, College of Medicine, University of Ibadan, Nigeria

**Keywords:** brain tumor, epidemiology, intracranial neoplasms, Nigeria, prevalence

## Abstract

**Background:**

Intracranial neoplasms, encompassing benign and malignant tumors, remain a significant health concern worldwide. This review systematically examines hospital-based reports of intracranial neoplasms in Nigeria from 1960 to July 2024, highlighting their distribution, demographics, and temporal trends.

**Methods:**

Following PRISMA guidelines, studies published from 1960 onward were identified through PubMed, Google Scholar, and African Journals Online. Eligible studies reported incidence, prevalence, or demographic data of intracranial neoplasms in Nigeria. Extracted data were pooled for meta-analysis to estimate aggregated incidence and prevalence.

**Results:**

Forty-five studies comprising 3517 cases were included. Pooled extrapolated prevalence was 3.22 (95% CI: 1.66-5.22), while compounded 10 yearly incidence was 3.66 (95% CI: 1.67-6.32). Reported incidence increased from 2.18% (1960-1969) to 4.08% (2000-2009) and 4.84% (2010-2019). Gliomas, meningiomas, and pituitary adenomas were the most frequent tumors. Among adults, meningioma predominated, followed by glioma and pituitary adenoma, whereas in children, gliomas were most common, followed by medulloblastoma and craniopharyngioma. Male predominance was observed (male-to-female ratio 1.17:1), with peak cases in the fourth to sixth decades. Regionally, southern Nigeria (southeast and southwest) showed higher incidence, reflecting disparities in diagnostic capacity and reporting compared with the north.

**Conclusion:**

Reported intracranial neoplasms in Nigeria have increased over the decades, with a prevalence of 3.22 and distinct demographic and geographic patterns. Strengthening diagnostic infrastructure, improving reporting systems, and establishing a national registry are essential for better understanding and management.

## Graphical Abstract

**Graphical Abstract:**
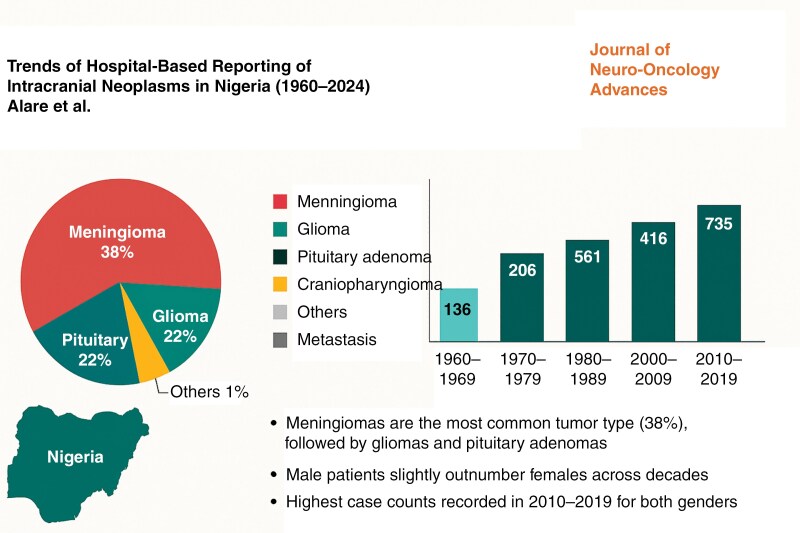

**Key Points:**

- Incidence of intracranial tumors in Nigeria rose significantly from 1960 to 2024.
- Gliomas, meningiomas, and pituitary adenomas were the most common tumor types.
- Southern Nigeria had higher prevalence due to better reporting of cases in literature.

**Importance of the Study**

This study is critical in highlighting the evolving epidemiology of intracranial neoplasms in Nigeria over 6 decades. By analyzing hospital-based records from 1960 to 2024, it provides valuable insight into the rising trend of reported brain tumors, likely reflecting improved diagnostics, increased awareness, and shifting population demographics. The identification of gliomas, meningiomas, and pituitary adenomas as the most common tumors, along with their age- and sex-specific patterns, enhances clinical understanding and guides targeted interventions. Importantly, the geographical disparities in incidence—especially the lower reports from northern regions—expose systemic inequalities in healthcare access, infrastructure, and reporting mechanisms. This underscores the urgent need for a national brain tumor registry, equitable distribution of diagnostic tools, and region-specific public health strategies. The findings serve as a foundational reference for policymakers, clinicians, and researchers aiming to reduce the burden and improve outcomes of intracranial neoplasms in Nigeria.

Worldwide, intracranial tumors lead to high rates of mortality and morbidity. The majority of these are primary brain tumors, and ~25%-50% are metastatic deposits from other body tissues.^1^ In addition to the variations in the types of intracranial tumors, the types of tumors vary with age. Clinically, proper characterization of tumors on the basis of their biological and histological properties is crucial in the development of treatment protocols. Likewise, epidemiologic data on intracranial tumors are essential to direct research and formulate policies. In many countries, data concerning cancer are retrieved from standardized cancer registries.^2^ These are crucial for monitoring trends, efficient intervention, and policy implementation.

In Nigeria, the first cancer registry began in 1960 in Ibadan and has since grown to include several other institutions and geographic locations within the country.^3^ However, despite efforts, there remains a deficit in the quality and scope of data collection.^4^ Coupled with the dearth of neurological services in Nigeria, this leads to a considerably less effective approach for managing these conditions and, consequently, relatively poorer patient outcomes.^5^ There are efforts by researchers to paint an epidemiological picture of intracranial tumors in Nigeria, but these efforts are not concerted and are largely inconsistent. Consequently, there is a need for an up-to-date picture of the epidemiology of intracranial tumors to help understand these trends and develop a public health response in managing this condition.

This systematic review aimed to describe an up-to-date trend in the hospital-based reports of intracranial tumors found in literature, highlighted trends in the incidence and types of tumors across different regions, and provided concise data to support further research in the subject area.

## Methods

### Study area

Among the nations that comprise Western Africa are Nigeria. Its 910,770 square kilometers provide accommodations for ~250 distinct ethnic groups.^6^ Together with a capital territory, its 36 states are split into 6 geopolitical zones or regions. Nigeria’s population is expected to be 228,973,667 (~229 million) as of Saturday, June 2024, on the basis of a Worldometer estimate based on United Nations data.^7^ With 2.78% of the world’s population, Nigeria may be regarded as the sixth most populated country in the world.^⁷^ The population is 43.4% younger than 14 years old. Individuals within the age range of 15-64 constitute 53.9% of the total population. The proportion of adults over 65 years of age is only 2.8%.^8^

### Protocol and registration

The current systematic review was completed in July 2024 in compliance with the Preferred Reporting Items for Systematic Review and Meta-Analyses (PRISMA) guidelines.^9,10^ The search was conducted after the protocol was accepted by the International Prospective Register of Systematic Reviews (PROSPERO) with the identification number CRD42024569796.

### Research question

The PICO framework was used for systematic reviews in line with the literature,^11^ as we aimed to assess data on the epidemiological distribution of intracranial neoplasms in Nigeria using hospital-based records

Population (P): Nigerians
Exposure (E): Intracranial neoplasms
Comparators (C): Not applicable
Outcomes (O): Prevalence and epidemiological distribution

Thus, our research questions were as follows:

1. What is the trends in epidemiological distribution of intracranial neoplasms in Nigeria and their variations across geopolitical zones using hospital-based records as reported in literatures?
2. What are the trends in the presentation and patterns of intracranial disease from 1960-2024?

The systematic review and meta-analysis were carried out via the Briggs Institute Reviewer manual approach, which consists of 5 steps: identifying the research question, discovering relevant papers, selecting studies, organizing data, and compiling, summarizing, and reporting the findings.^12^

### Search strategy

A comprehensive literature search was conducted across multiple databases, including PubMed, SCOPUS, Google Scholar, and AJOL. The search strategy was designed to include terms related to epidemiology distributions of intracranial neoplasms in Nigeria. The reference lists of the included studies and relevant reviews were manually searched to identify additional studies. The search terms used for each database are reported in Supplementary File 1.

### Eligibility criteria

Studies were included in the review if they met the following criteria: 1) hospital-based research with information on the prevalence, potential predisposing factors, and treatment outcomes of intracranial neoplasms; 2) studies in which the full text was published in the English language; and 3) original research, cross-sectional, cohort, and case-control studies.

We excluded studies that 1) were solely based on a self-reported diagnosis of intracranial neoplasms; 2) were studies in which a clear diagnosis of intracranial neoplasms was not made; and 3) case series, reviews, commentaries, expert opinions, conference proceedings, letters, or editorials.

### Study selection

**Figure 1.**
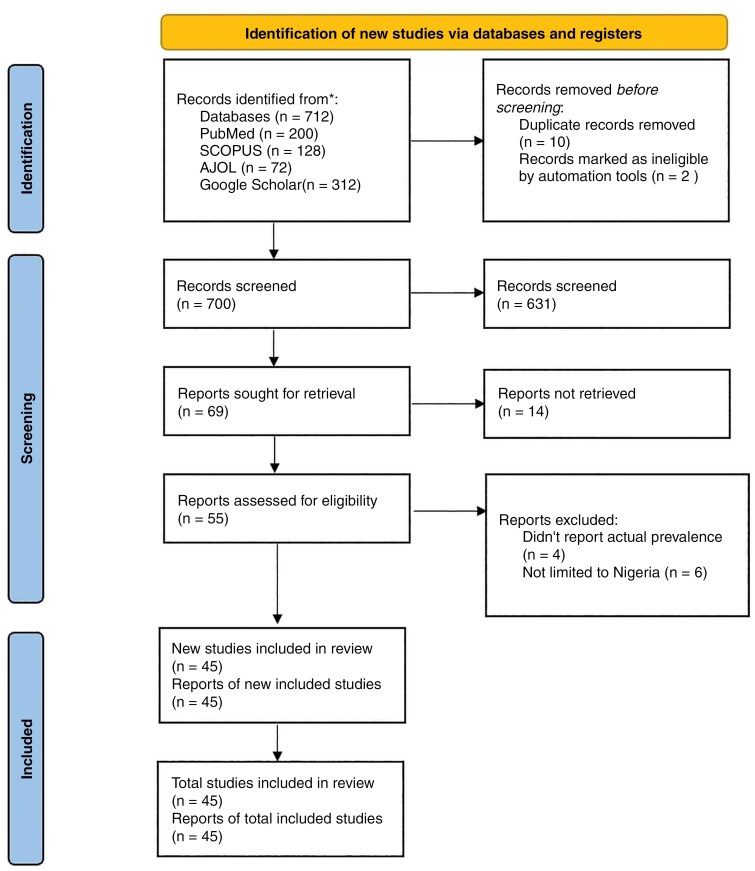
PRISMA flow chart.

Mendeley (London, UK; Elsevier) removed duplicates. Using Rayyan software, 3 pairs of reviewers (B.A., C.E.C., E.K.O., K.A.P., O.O., and P.A.) independently reviewed the abstracts. The third reviewer (K.A., S.I.A., and N.D.B.) resolved any discrepancies between the 2 reviewrs’ evaluations. Full texts of research that might be relevant were assessed. Throughout all the methodological stages, the agreement rate between the 2 reviewers was assessed via Cohen’s kappa coefficient. A score of 0.83 was considered to indicate “strong agreement.” The process is summarized in the PRISMA chart in Figure 1.

### Data extraction

Together, the 2 reviewers searched the literature and assessed pertinent research, ensuring that the selection criteria were consistently adhered to by employing an eligibility criterion. The study’s location, length, year of publication, design, study setting (rural or urban), sample size, diagnostic standards, and population mean age were all obtained. The retrieved data were stored in Microsoft Excel file format and organized according to Nigeria's geopolitical zones for a methodical assessment.

### Data analysis

Meta-analysis was done for the prevalence of intracranial neoplasms in Nigeria, 10-yearly cumulative incidence from 1960 to June 2024, Geopolitical Zone distribution, frequency of occurrence of different types of intracranial neoplasms among adults and pediatric group, and also gender distribution of intracranial neoplasm using STATA (version 14.0, Stata Corporation, Texas, USA).

### Quality assessment

The following assessment criteria are part of the Newcastle‒Ottawa Scale (NOS), which was used to determine the quality of the research: nonresponse rate, representativeness of the cases, sufficient case definition, and identical ascertainment method.^13^ The NOS’s total quality assessment score varied from 0 stars, which represents the lowest quality, to 9 stars, which represents the best quality.^14^ Studies that received seven stars or higher were usually considered to be of high quality.^15^ The selected articles ranged in quality from intermediate to high.

## Results

A total of 712 articles were identified through the literature search. Mendeley (Elsevier, London, UK) was used to exclude the 12 papers that were determined to be duplicates, leaving 700 original studies. Following the analysis of the 700 titles and abstracts, the reviewers eliminated 10 of the entries due to issues with reporting the true prevalence, and several of the studies contained information from neighboring nations. In the subsequent stage, the reviewers examined 616 complete texts while taking the inclusion and exclusion criteria for research on prevalence into account. Finally, this pooled analysis included 45 studies with 377,856 cases (Table 1).

**Table 1:** Characteristics of included studies

Author	Year	Enrollment period	State(s)	Geopolitical Zone	Population Group	Types of study	Sample Size	Prevalence %	Male to Female ratio	Mean age (year)	Primary/ Metastasis	Prevalence per types
Balogun^16^	2022	2010–2019	Oyo, Lagos, Enugu, Plateau, Kaduna	South West; South East; North Central, North West	Adolescents and Young adults	Multicenter, retrospective cross-sectional	104	NR	57:47	NR	Primary	Glioma (29.8%) Pituitary Adenoma and Craniopharyngioma (30.8%)
Ndubuisi^17^	2018	2006–2017	Enugu	South East	Pediatrics	Retrospective longitudinal study	54	NR	NR	NR	Mixed	Glioma (37%) Medulloblastoma (24.1%) Meningioma (3.1%)
Osaguona^18^	2020	2013–2020	Edo	South South	Papilloedemic patients	Retrospective case-control	66	12.1%	23:43	36.9 ± 15.3	NR	NR
Nwokoro^19^	2023	2008–2017	Enugu	South East	Adults	Retrospective longitudinal	NR	NR	55:45	45.3	Primary	Pituitary Adenoma (19.6%) Meningioma (38.9%) Glioma (30.9%) Craniopharyngioma (3.5%) Medulloblastoma (2.5%) Hemangioblastoma (1.6%)
Ndubuisi^20^	2017	2006–2015	Enugu	South East	NR	Retrospective longitudinal	252	41.2%	137:115	42.8	Mixed	Meningioma (32.9%) Glioma (23.8%) Pituitary Adenoma (13.5%) Craniopharyngioma (7.5%) Metastasis (5.6%) Vestibular Schwannoma (1.2%)
Idowu^21^	2007	1999–2004	Oyo	South West	NR	Retrospective longitudinal	113	NR	62:51		Primary	Gliomas (23%), meningiomas (23%), pituitary adenoma (16.8%), craniopharyngiomas (15.9%), and medulloblastoma (12.4%)
Igun^22^	2001	1989–1998	Plateau	North Central	NR	Retrospective analysis	30	3%	NR	NR	Mixed	Metastatic (30%) Pituitary Adenoma (21%) Meningioma (18%)
Jibrin^23^	2018	2005–2015	FCT	North Central	NR	Retrospective longitudinal	20,191 Case: 121	0.5%	NR	35 ± 17.1	Mixed	Meningioma (41%) Pituitary Adenoma (22%) Astrocytoma/glioma (20%)
Udoh^24^	2022	2007–2020	Edo	South South	Pediatrics	Retrospective longitudinal study	178	20.22%	90:88		Primary	
Ndubuisi^25^	2021	2016–2018	Enugu, Abia, Ebonyi	South East	Children with brain tumor	Prospective observational study	29	NR	14:15	7. 93 ± 4.21	Primary	Cerebellar tumor (51.7%) Medulloblastoma (13.8%) Craniopharyngioma (10.3%)
Ogun^26^	2016	2001–2010	Oyo	South West	Children	Retrospective longitudinal	77	2.5%	44:33	7.2	Primary	Astrocytoma (20%) Ependymomas (19.5%) Medulloblastoma (16.9%) Craniopharyngioma (11.7%) Glioma (39%)
Mezue^27^	2012	2002–2010	Enugu	South East	NR	Retrospective longitudinal	68		33:35		Primary	Meningioma (23.8%)
Uchime^28^	2023	2008–2017	Lagos	South West	NR	Hospital-based Retrospective	296	1.06%	131: 165	37.0	Mixed	Meningioma (35%) Pituitary Adenoma (26%) Glioma (24%)
Olasode^29^	2016	2001–2011	Osun	South West	Pediatric	Hospital-based retrospective	111 Case: 13	11.7%	56:53		Primary	Astrocytoma (38.5%) Glioma (46.2%) Choroid plexus papilloma (15.4%)
Danjuma^30^	2022	2016–2019	Kaduna	North West	Adult	Hospital-based Retrospective	39		21:18	49.8 ± 11.8	Mixed	Pituitary Adenoma (35.9%) Glioma (23.1%) Meningioma (15.4%) Metastasis (15.4%)
Adeloye^31^	1968	—	Oyo	Southwest	NR	Hospital-based Retrospective	269 Case: 21	7.8%	149:120		Mixed	Primary tumor: 19% Metastasis: 81%
Ali^32^	2019	2016–2018	Bornu	North East	NR	Hospital-based Retrospective	40		17:23	28.4 ± 20.	Mixed	Meningioma (20%) Metastatic (15%) Glioma (12.5%) Astrocytoma (10%) Craniopharyngioma (10%) Hemangioma (5%)
Soyemi^33^	2015	2008–2012	Lagos	South West	NR	Hospital-based Retrospective	12,610 cases: 56	0.4%	27:29	36 ± 20.35	Primary	Gliomas (39.3%) Astrocytoma (30%) Meningioma (29%) Medulloblastoma (18%) Pituitary Adenoma (14%) Ependymomas (9%)
William^34^	1975	1960–1972	Oyo	South West	Children	Cancer Registry Retrospective	1352 Case: 29	2.2%	17:12	NR	Primary	Glioma (48.3%) Astrocytoma (37.9%) Craniopharyngioma (24.1%) Medulloblastoma (10.3%) Ependymoma (6.8%)
Ezeala-Adikaibe^35^	2017	2003–2013	Enugu	South East	NR	Hospital-based Retrospective	196 Case: 40	20.4%	135:61	46.8 ± 18.6	NR	NR
Awodele^36^	2011	2005–2009	Oyo and Lagos	South West	NR	Cancer Registries based Retrospective	5094 Case: 199	3.9% Lagos: 3.8% Oyo: 4%	109:90	NR	NR	NR
Olasode^37^	2000	1980–1990	Oyo	South West	NR	Hospital-based Retrospective	210		105:105	NR	Mixed	Glioma (29.5%) Metastatic (22.8%) Astrocytoma (21%) Pituitary Adenoma (17.1%) Meningioma (11.4%) Craniopharyngioma (9.0%) Hemangioma (4.8%) Glioblastoma multiforme (4.3%) Oligodendroma (3.8%) Medulloblastoma (3.8%)
Adewuy^38^i	2013	2006–2010	Kaduna	North West	Pediatric	Hospital-based Retrospective	136 Case: 6	4.4%	77:59	6.9	NR	NR
Osuntokun^39^	1971	1957–1969	Oyo	South West	NR	Hospital-based Retrospective	220;000 Case: 108	0.05%		NR	Mixed	Glioma (33.3%) Metastatic (26%) Meningioma (18.5%)
Afolayan^40^	2008	1992–1996	Kaduna	North West	NR	Cancer Registry-based Retrospective	1887 Case: 4	0.21%	2:2	NR	NR	NR
Aghadiuno^41^	1985	1960–1982	Oyo	South West	Children	Hospital-based Retrospective	89	NR	59:30	NR	Mixed	Glioma (46.1%) Astrocytoma (39%) Metastatic (23.6%) Medulloblastoma (13%) Craniopharyngioma (9%) Ependymoma (4%)
Adeloye^42^	1982	1973–1979	Oyo	South West	NR	Hospital-based Retrospective	213	NR	NR	NR	Mixed	Pituitary adenoma (29%) Metastatic (24%) Glioma (22%) Meningioma (17%)
Seleye-Fabura^43^	2004	1990–2001	Rivers	South South	Children	Hospital-based Retrospective	173 Case: 1	0.6%	NR	NR	Primary	Medulloblastoma (100%)
Sahabi^44^	2017	2006–2015	Sokoto	North West	Children	Hospital-based Retrospective	358 Case: 10	2.8%	7:3	NR	Primary	Medulloblastoma (80%) Hemangioblastoma (20%)
Shehu^45^	2012	2001–2010	Kano and Osun	North West South West	Children	Hospital (multicenter)-based Retrospective	Kano 410 Case: 5 Osun 568 Case: 3	Kano: 1.2% Osun: 0.5%	Kano: 3:2 Osun: 3:0	NR	Mixed	NR
Akang^46^	1996	1973–1990	Oyo	South West	Children	Hospital-based Retrospective	1881 Case: 142	7.5%	74:68	NR	NR	Astrocytoma/Glioma (39%)
Utuk^47^	2015	2007–2014	Akwa-Ibom	South South	Children	Hospital-based Retrospective	84 Case 2	2.3%	0:2	NR	NR	NR
Babatunde^48^	2015	1991–2010	Oyo	South West	Children	Hospital-based Retrospective	625 Case: 43	6.9%	20:23	7.22 ± 3.71	NR	NR
Akinsete^49^	2018	2015–2017	Lagos	South West	Children	Hospital-based Retrospective	179 Case: 3	1.6%	NR	5	NR	NR
Ahmad^50^	2016	2006–2013	Kaduna	North West	Children	Hospital-based Retrospective	426 Case: 9	2.1%	NR	NR	NR	NR
Eke^51^	2021	2011–2019	Rivers	South South	Children	Hospital-based Retrospective	266 Case: 6	2.3%	NR	NR	NR	NR
Sahabi^52^	2016	1991–2007	Oyo	South West	NR	Hospital-based Retrospective	91,842 Case:419	0.5%	204:152	32.7 ± 18.9	Mixed	Glioma (40.2%) Astrocytoma (24.7%) Meningioma (23.6%) Pituitary Adenoma (14%) Craniopharyngioma (7.9%)
OHAEGBULAM^53^	1980	1974–1979	Enugu	South East	NR	Hospital-based Retrospective	48				Mixed	Glioma (20.8%) Pituitary Adenoma and Craniopharyngioma (18.8%) Meningioma (16.7%) Metastatic (10.4%)
Sahabi^54^	2019	2008–2017	Sokoto	North West	NR	Hospital-based Retrospective	151		92:59	28.17 ±17.26	Mixed	Meningioma (37.9%) Astrocytoma/Glioma (23.2%) Craniopharyngioma (11.9%) Pituitary Adenoma (7.3%)
Soyemi^55^	2020	2013–2018	Lagos	South West	NR	Hospital-based Retrospective	13,651 Case:113	0.82%	54:59	45 ± 23.6		Meningioma (47.8%)
Fapohunda^56^	2020	2015–2018	Lagos	South West	NR	Hospital-based Retrospective	546 Case: 4	0.73%	2:2			
Uchedu^57^	2020	2014–2019	Delta	South South	NR	Hospital-based Retrospective	668 Case:7	1.1%	5:2	49		
Owolabi^58^	2010	2005–2008	Kano	North West	Neurological patients	Hospital-based Retrospective	980 Case: 18	1.8%	9:9			
Ogunleye^59^	2023	2018–2021	Bauchi	North East	Neurosurgical patients	Hospital-based Retrospective	1713 Case: 27	1.6%				
Umar^60^	2021	2015–2018	Bornu	North East	Stroke Patients	Hospital-based Retrospective	138 Case: 4	2.9%	3:1			

NR: Not reported.

### Description of included studies

**Figure 2.**
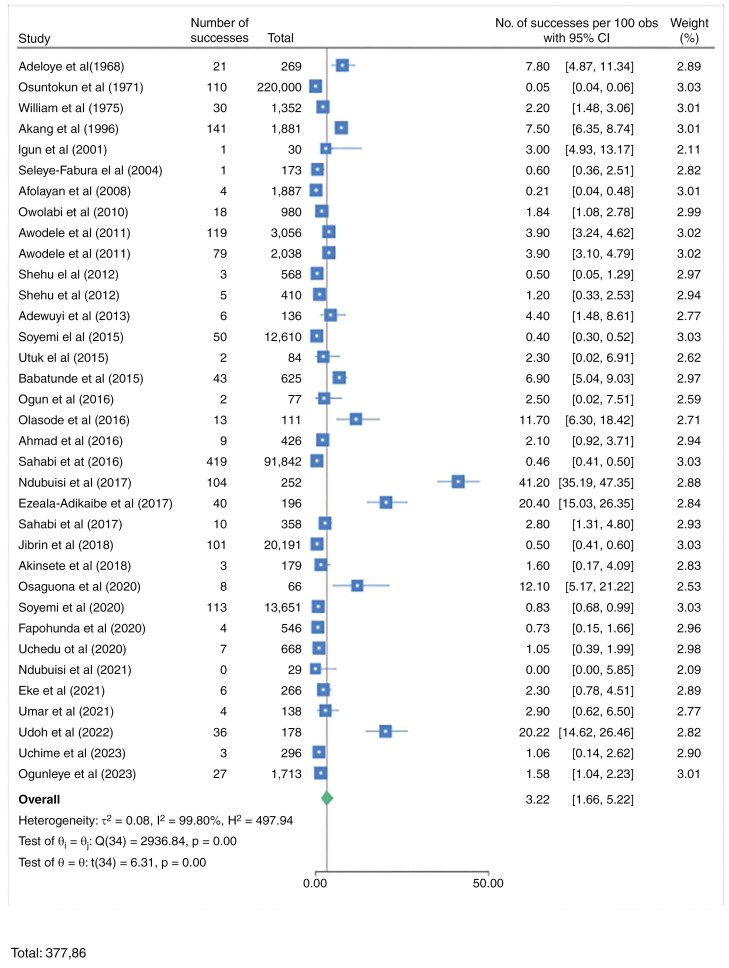
Forest plot of the incidence of intracranial neoplasms.

The included studies are predominantly retrospective in design, conducted in various geopolitical zones in Nigeria, with enrollment periods ranging from the 1960s to 2021.^16–60^ The studies span multiple states, including Oyo, Lagos, Enugu, Kaduna, Plateau, Rivers, and others, reflecting a broad geographical distribution across the southwestern, southeastern, northwestern, and northeastern regions among others, as shown in Figure 2. Populations studied vary by age group, with a focus on pediatric, adolescent, young adult, and adult populations. Many studies target specific patient groups, such as those with brain tumors or papilloedema. Geographical distribution of included studies is shown in supplemental Figure 1.

The types of studies conducted are mostly retrospective longitudinal or hospital-based cross-sectional studies, with some prospective observational studies. The sample sizes vary widely, ranging from as few as 4 patients in rare cases to over 20 000 in broader, hospital-based registries. The incidence rates and male-to-female ratios are inconsistently reported across studies, but there is often a slight male predominance. The mean ages of the study populations also vary, with pediatric populations showing a lower mean age (~7-10 y) and adult populations having higher mean ages (typically ~35-50 y).

These studies focused on both primary and metastatic brain tumors, with gliomas, astrocytomas, and meningiomas being the most common tumor types across the studies. Pediatric studies frequently report gliomas and medulloblastomas, whereas adult studies often cite gliomas, meningiomas, and pituitary adenomas. The prevalence of specific tumor types varies, with gliomas often accounting for a substantial proportion of primary brain tumors, and metastatic tumors being less common but notable in certain regions. Overall, these studies provide a comprehensive overview of brain tumor epidemiology in Nigeria, highlighting regional and age-specific differences in tumor prevalence.

### Outcome measures

#### Extrapolated prevalence rate

**Figure 3.**
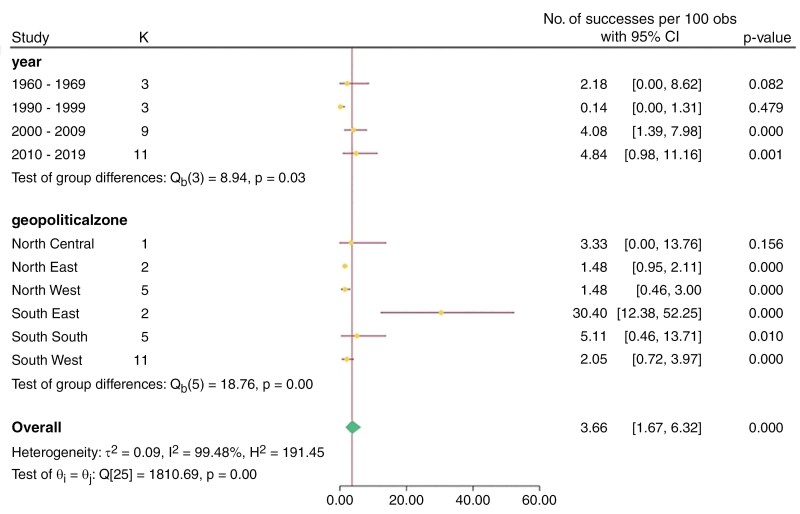
Forest plot of 10-y cumulative incidence showing geopolitical distributions.

The extrapolated pooled hospital-based records prevalence found in literature from 1960 to2024 was 3.22% (95% CI: 1.66-5.22), whereas the extrapolated pooled 10-year cumulative incidence was 3.66% (95% CI: 1.67-6.32); the incidence rose from 2.18% between 1960 and 1969 to 4.08% between 2000 and 2009 and eventually to 4.84% between 2010 and 2019. In terms of the geographical distributions of intracranial neoplasm occurrence, the Southeast Region of Nigeria had the highest pooled prevalence, 16.81%; (95% CI 0.24-48.25); South‒South, 4.6%; (95% CI 0.68-11.30); and North Central had the lowest pooled prevalence at 0.56%. In the subanalysis of the 10-year cumulative incidence according to politics, Southeast also had the highest value of 30.40% (95% CI 12.38-52.25), whereas Northwest and Northeast had the lowest value of 1.48%. All the data are shown in Figures 2 and 3 and Table 2. Supplemental figure 2 showed rise in 10-yearly prevalence of intracranial neoplasms.

**Table 2:** Prevalence of intracranial neoplasms across geopolitical zones

Geopolitical Zone	Number Studies	Prevalence (%)	95% Confidence Interval	*I*² Statistics
**North-Central**	**2**	**0.56**	**0–4.75**	**57.97**
**North-East**	**2**	**1.72**	**0.73–3.07**	**32.29**
**North-West**	**6**	**1.65**	**0.65–3.04**	**86.07**
**South-East**	**3**	**16.81**	**0.24–48.25**	**97.86**
**South-South**	**6**	**4.6**	**0.68–11.30**	**94.77**
**South-West**	**16**	**2.33**	**1.10–3.97**	**99.76**

### Gender distribution

There is a slight male predominance of intracranial neoplasms with a male-to-female ratio of 1.17:1. The 10 yearly distributions since 1960 show that males have always had a slight predominance of intracranial neoplasms, with male-to-female ratios of 1.2:1 between 1960 and 1969, 1.03:1 between 1980 and 1989, 1.3:1 between 1990 and 1999, 1.3:1 between 2000 and 2009, and 1.03:1 between 2010 and 2019, as shown in Table 3. Gender distribution of intracranial neoplasms across different decades is graphically presented in supplemental Figure 3.

**Table 3.** Frequency of distributions of gender and tumor types

Category/Type	Number/Percentage
Gender distribution across all decades	
1960–1969	
Male	166
Female	142
1970–1979	
Male	—
Female	—
1980–1989	
Male	179
Female	173
1990–1999	
Male	206
Female	154
2000–2009	
Male	551
Female	416
2010–2019	
Male	735
Female	716
Overall distributions of Intracranial Neoplasms	
Glioma	32%
Meningioma	27%
Pituitary adenoma	15%
Metastasis	8%
Craniopharyngioma	5%
Medulloblastoma	3.3%
Others (Hemangioma, Hemangioblastoma, Choroid plexus papilloma, Vestibular schwannoma, Oligodendroglioma)	9%
Distributions of Gliomas	
Astrocytoma	87%
Ependymoma	12%
Glioblastoma multiforme	1%
Distributions of intracranial neoplasms in adult population	
Meningioma	38%
Glioma	32%
Pituitary adenoma	22%
Craniopharyngioma	3.4%
Metastasis	1.1%
Others (Medulloblastoma, Hemangioma)	4%
Distributions of intracranial neoplasms in pediatric population	
Glioma	61%
Medulloblastoma	20%
Craniopharyngioma	10%
Metastasis	8%
Others (Hemangioma, Hemangioblastoma, Choroid plexus papilloma, Vestibular schwannoma, Oligodendroglioma)	2.2%

Others: Hemangioma, Hemangioblastoma, Choroid plexus papilloma, Vestibular schwannoma, Oligodendroma.

### Distributions of different types of intracranial neoplasms

This study revealed that, overall, gliomas are the most common intracranial neoplasms in Nigeria, accounting for 32% of all brain tumors, followed by meningioma, accounting for 27%, and pituitary adenoma, accounting for 15%, as shown in Table 3. Table 3 shows the distributions of gliomas, with astrocytoma being the most common type, accounting for 87% of all gliomas. However, meningioma is the most common intracranial neoplasm among adult populations, followed by gliomas and pituitary adenoma, as shown in Table 3, whereas gliomas are the most common intracranial neoplasm among pediatric populations, followed by medulloblastoma and craniopharyngioma, as shown in Table 3. Distribution of various types of intracranial neoplasms across different age groups are shown in supplemental figures 4, 5, 6, and 7.

## Discussion

This systematic review of intracranial neoplasms in Nigeria from 1960 to July 2024 highlights several important epidemiological trends and highlights the burden of these tumors in the country. This study revealed an increasing number of cases of intracranial neoplasms reported in literature over the decades, with the extrapolated reported incidence increasing from 2.18% in the 1960s to 4.84% in the 2010s. This suggests improved diagnostic capabilities over time, heightened awareness among healthcare professionals, and perhaps changes in environmental or genetic risk factors. However, the overall increase in incidence may also reflect population growth and aging, as intracranial neoplasms typically manifest in middle-aged and older adults, particularly in the fourth to sixth decades of life.^61–63^

The findings of this study regarding the most common tumor types align with global patterns. Gliomas, meningiomas, and pituitary adenomas are the dominant intracranial neoplasms, with meningiomas being the most common in adults.^64–67,68^ This contrasts with the pediatric population, where gliomas, medulloblastomas, and craniopharyngiomas are more prevalent.^69–72,68^ This finding is also similar to the findings of Ekpeng et al. in Ghana, who reported that astrocytoma is the most common form of glioma in Ghana, which is similar to what we found in Nigeria.^73^ This demographic division of tumor types is consistent with known tumor biology, where meningiomas, which are often benign, occur more frequently in adults, whereas pediatric brain tumors tend to be more aggressive.^74–76^

A key observation is the geographic disparity in intracranial neoplasm cases, with higher extrapolated incidences reported in southern Nigeria, particularly in southeastern and southwestern Nigeria. This disparity could be attributed to the number of solid academic centers in the southern Nigeria contributing increase number of research output and also better diagnostic infrastructure, such as the availability of neuroimaging modalities, more urbanized and economically developed regions and reduced research output on cases of intracranial neoplasm in the northern part of the country. Access to neurosurgical services and expertise may also be more concentrated in southern Nigeria, resulting in higher detection and reporting rates. In contrast, northern Nigeria, which may face infrastructural and healthcare resource challenges, could underreport cases, leading to an apparent lower incidence. The uneven distribution of cases highlights the need for equitable healthcare development and the establishment of advanced neurosurgical and diagnostic centers across all regions.

The male predominance in the incidence of intracranial neoplasms, with a male-to-female ratio of 1.17:1, is noteworthy. This pattern is observed globally in certain brain tumors such as gliomas, although some other neoplasms, such as meningiomas, often show a female preponderance.^73,68,77,78^ The reasons for this sex difference in Nigeria could warrant further investigation, particularly in exploring genetic, hormonal, and environmental factors.

### Conclusion

An extensive investigation of the epidemiological distribution and trends of intracranial neoplasms in Nigeria over a six-decade period is presented in this systematic review. Although, cancer registries and population-based studies will give better picture to the actual prevalence and incidence of intracranial neoplasms in Nigeria, but lack of these studies does not mean that intracranial neoplasms are not reported in the country making studies that used hospital-based records necessary at this point. The results revealed a consistent increase in the incidence of these malignancies, which may be due to changes in population dynamics, increased awareness, and advancements in diagnostic capability. The study suggests clear demographic trends, with a higher prevalence in men. The most prevalent tumor types are gliomas, meningiomas, and pituitary adenoma, with gliomas being more common in children and meningiomas leading in adults.

The disparity in the occurrence of the disease between the northern and southern parts of the country emphasizes the necessity of setting up specialist diagnostic and treatment facilities across the nation as well as the equitable distribution of medical resources. To better understand the burden of intracranial neoplasms and improve outcomes, this study recommends the establishment of a national registry for brain tumors as well as prospective studies. These endeavors, in conjunction with expanded public health programs, are essential for addressing the increasing prevalence and controlling the expanding load of intracranial neoplasms in Nigeria.

This review underscores the growing burden of intracranial neoplasms in Nigeria and calls for improved national-level diagnostic capabilities, public health policies, and the creation of a national brain tumor registry. These steps are critical for managing the increasing incidence of these neoplasms and improving patient outcomes.

### Strengths and limitations of the study

The substantial number of participants in this comprehensive literature review and meta-analysis is the study’s strongest point. Furthermore, the included studies encompass all 6 geopolitical regions of Nigeria, making it possible to identify regional variations in the incidence of intracranial neoplasms. The primary constraint of this research is the fact that the methodology meta-analysis of hospital-based records cannot be used to determine the actual prevalence and incidence of intracranial neoplasms in Nigeria but rather gives a clue to the true picture which number might be much higher than what presented here. Also, uneven allocation of included studies among the geopolitical zones; nonetheless, since there are publications from every geopolitical zone, deductions can be drawn. We will recommends funding of nationwide population-based studies that will look into the actual prevalence of intracranial neoplasms in the country and also optimizing and synchronizing cancer registries to be able to provide valid data for this kind of study in the future.

## Supplementary material

Supplementary material is available online at *Neuro-Oncology Advances* (https://academic.oup.com/noa).

vdaf195_suppl_Supplementary_Figures_1-7

vdaf195_suppl_Supplementary_Table_S1
