# Supplementary figures and images for: Trends of hospital-based reporting of intracranial neoplasms in Nigeria from 1960 to 2024: A systematic review and pooled analysis of literatures
Source: Neurooncol Adv. 2025 Sep 2;7(1):vdaf195. doi: 10.1093/noajnl/vdaf195 (PMC12658747; doi:10.1093/noajnl/vdaf195)

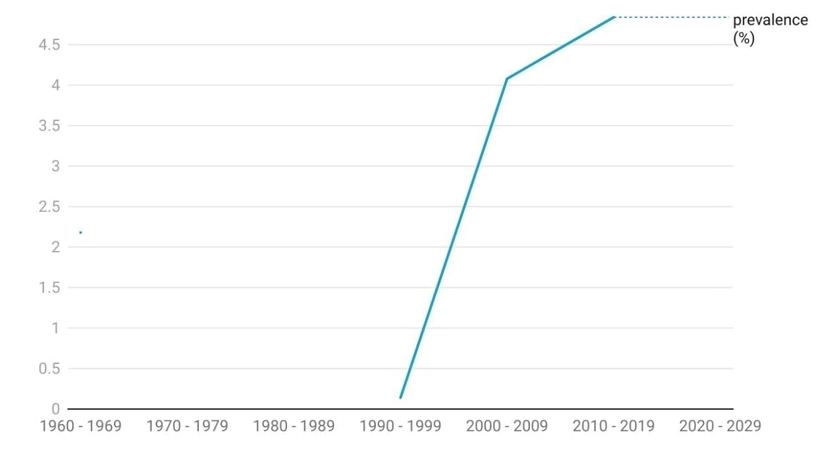

Supplement: vdaf195_suppl_Supplementary_Figures_1-7 [file vdaf195_suppl_supplementary_figures_1-7.zip › vdaf195_suppl_Supplementary_Figuress_1-7/image_1.tiff]

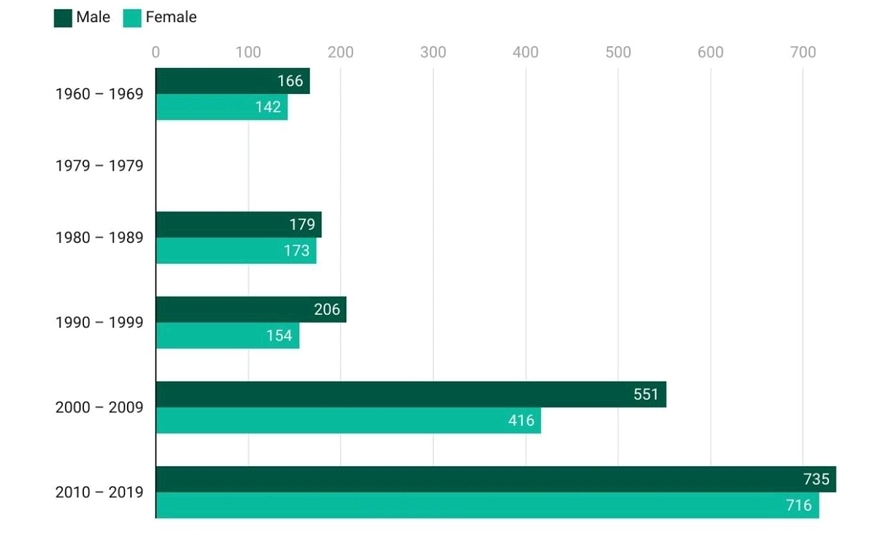

Supplement: vdaf195_suppl_Supplementary_Figures_1-7 [file vdaf195_suppl_supplementary_figures_1-7.zip › vdaf195_suppl_Supplementary_Figuress_1-7/image_10.tiff]

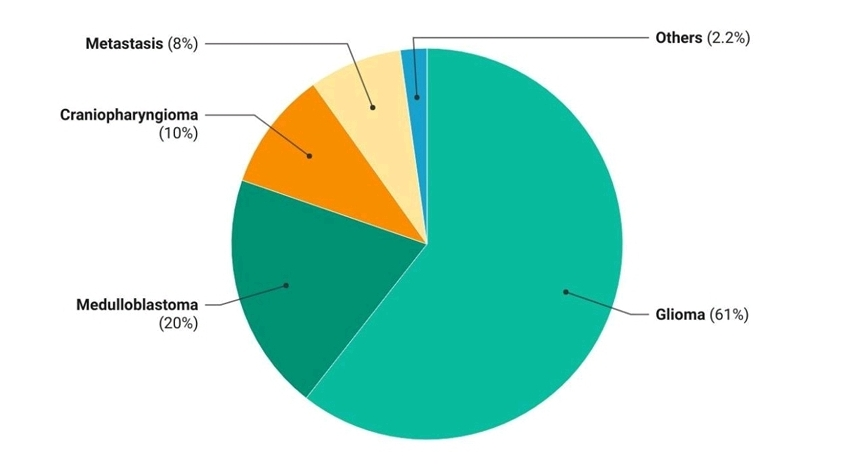

Supplement: vdaf195_suppl_Supplementary_Figures_1-7 [file vdaf195_suppl_supplementary_figures_1-7.zip › vdaf195_suppl_Supplementary_Figuress_1-7/image_2.tiff]

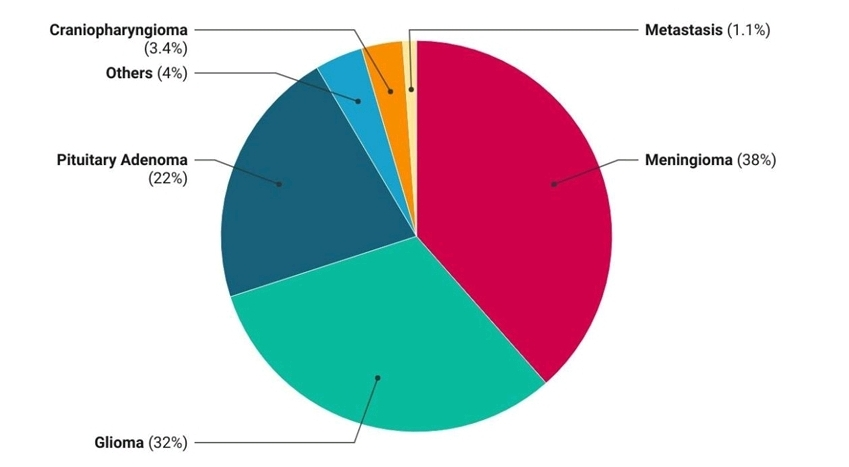

Supplement: vdaf195_suppl_Supplementary_Figures_1-7 [file vdaf195_suppl_supplementary_figures_1-7.zip › vdaf195_suppl_Supplementary_Figuress_1-7/image_4.tiff]

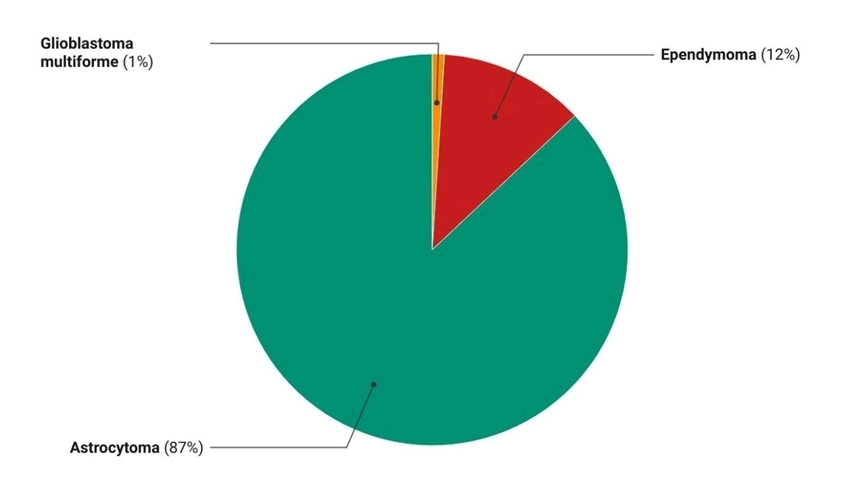

Supplement: vdaf195_suppl_Supplementary_Figures_1-7 [file vdaf195_suppl_supplementary_figures_1-7.zip › vdaf195_suppl_Supplementary_Figuress_1-7/image_6.tiff]

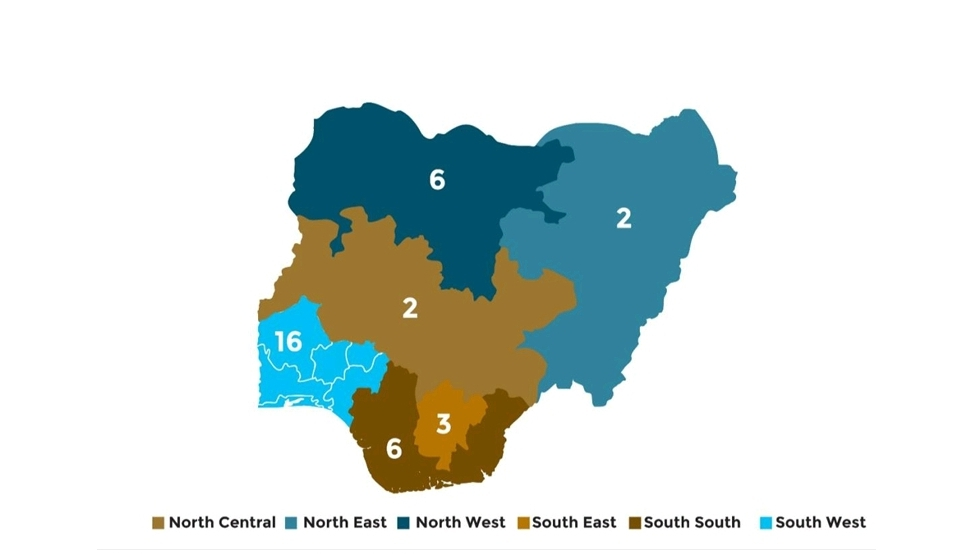

Supplement: vdaf195_suppl_Supplementary_Figures_1-7 [file vdaf195_suppl_supplementary_figures_1-7.zip › vdaf195_suppl_Supplementary_Figuress_1-7/image_7.tiff]

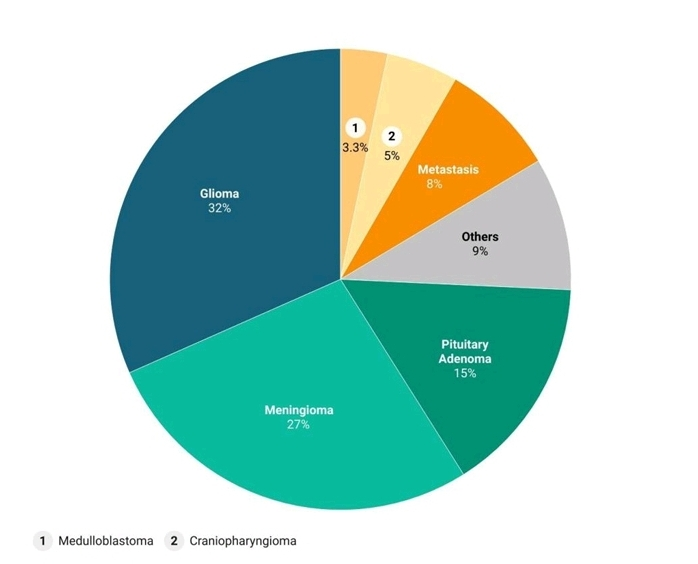

Supplement: vdaf195_suppl_Supplementary_Figures_1-7 [file vdaf195_suppl_supplementary_figures_1-7.zip › vdaf195_suppl_Supplementary_Figuress_1-7/image_8.tiff]
