# Supplementary material for: Trends of hospital-based reporting of intracranial neoplasms in Nigeria from 1960 to 2024: A systematic review and pooled analysis of literatures
Source: Neurooncol Adv. 2025 Sep 2;7(1):vdaf195. doi: 10.1093/noajnl/vdaf195 (PMC12658747; doi:10.1093/noajnl/vdaf195)
Supplement: vdaf195_suppl_Supplementary_Table_S1 [file vdaf195_suppl_supplementary_table_s1.docx]

| **Search Strings** | |
| --- | --- |
| PubMed | ("Intracranial Neoplasms"[MeSH]) AND ("Epidemiology"[MeSH Subheading] OR "Incidence"[MeSH] OR "Prevalence"[MeSH]) AND ("Nigeria"[MeSH]) AND ("1960"[DP] : "2024"[DP]) |
| AJOL | ("intracranial neoplasms" OR "brain tumors" OR "central nervous system tumors")  AND  ("epidemiology" OR "incidence" OR "prevalence" OR "distribution" OR "risk factors")  AND  ("Nigeria")  AND  ("1960" OR "1970" OR "1980" OR "1990" OR "2000" OR "2010" OR "2020") |
| SCOPUS | (TITLE-ABS-KEY("intracranial neoplasms" OR "brain tumors" OR "central nervous system tumors"))  AND (TITLE-ABS-KEY("epidemiology" OR "incidence" OR "prevalence" OR "distribution" OR "risk factors"))  AND (TITLE-ABS-KEY("Nigeria"))  AND (PUBYEAR > 1959) |
| Google Scholar | "Intracranial neoplasms" OR "brain tumors" OR "central nervous system tumors" AND "epidemiology" OR "incidence" OR "prevalence" AND "Nigeria" |

Table1Supplement: Search Strings per Databases
